# The role of immunoproteasomes in modulating neuro‐inflammation in Alzheimer’s Disease

**DOI:** 10.1002/alz.086577

**Published:** 2025-01-03

**Authors:** Mayank Kumar, Shan Jiang, Daniella Lorman, Larr David, Rajesh Soni, Michael Kissner, Mu Yang, Natura Myeku

**Affiliations:** ^1^ Columbia University Medical Center, New York, NY USA; ^2^ Columbia University Medical Center, NEW YORK, NY, NY USA

## Abstract

**Background:**

The immunoproteasome (IP) is the inducible form of the constitutive 20S proteasome upregulated in immune cells. The IP consists of 3 inducible β subunits (β1i – LMP2, β2i – MECL1, and β5i – LMP7), which generate MHC‐I compatible peptides. IPs are induced by pro‐inflammatory cytokine signaling, as well as oxidative stress signaling. Furthermore, in AD, the presence of aβ results in induction of IP in glial cells like microglia and astrocytes. The evidence for the induction of IPs in Alzheimer’s Disease (AD) pathology is numerous and unequivocal; however, the mechanism of induction of IPs in AD is still unclear. Accordingly, this study adds evidence to answer the question of whether IPs are induced as a compensatory mechanism for restoring homeostasis in AD or are induced due to the vicious cycle of inflammation and add to its pathological consequences.

**Method:**

The mice used in the study were genetically modified from a C57/BL6J background to obtain an AD model. This was achieved by knock‐in (KI) of Amyloid Precursor Protein (APP^NL‐G‐F/NL‐G‐F^) gene, and human MAPT (hTau^+/+^) gene. Furthermore, to obtain IP deficient mice, the KI mice had the lmp7^‐/‐^ (βi5) and mecl^‐/‐^ (βi2) genes knocked out.

Quantitative analysis of confocal images was performed for AD progression and gliosis levels (n = 4). This was correlated with functional changes of Affective and Cognitive behaviors (n = 10). To observe changes in immune response and neuro‐inflammation, multi‐color FACS (n = 3), subsequent bulk RNA sequencing (n = 3), along with snRNAseq (n = 3) was performed for microglia, lymphocytes, and astrocytes. In addition, multiplex cytokine assay (n = 3) was performed for gauging the neuro‐inflammation along with proteomics of CSF (n = 6), and immunopeptidomes (n = 3) of MHC‐I &II peptides by Mass Spectrometry.

**Result:**

It was observed that IP deficiency caused a distinct change in microglial and astrocytic phenotypes and an increased infiltration of lymphocytes. This was seen to increase pro‐inflammatory cytokines in IP deficient mice with a decrease in novel MHC associated immune peptides.

**Conclusion:**

It can be concluded that IP deficiency causes increased inflammation, gliosis, and consequent progression of AD.

